# Supplementary material for: TCF12 and LncRNA MALAT1 Cooperatively Harness High Cyclin D1 but Low β-Catenin Gene Expression to Exacerbate Colorectal Cancer Prognosis Independently of Metastasis
Source: Cells. 2024 Dec 10;13(24):2035. doi: 10.3390/cells13242035 (PMC11674632; doi:10.3390/cells13242035)
Supplement: Supplementary file 1 [file cells-13-02035-s001.zip › cells-3334694-supplementary.pdf]

Supplemental File

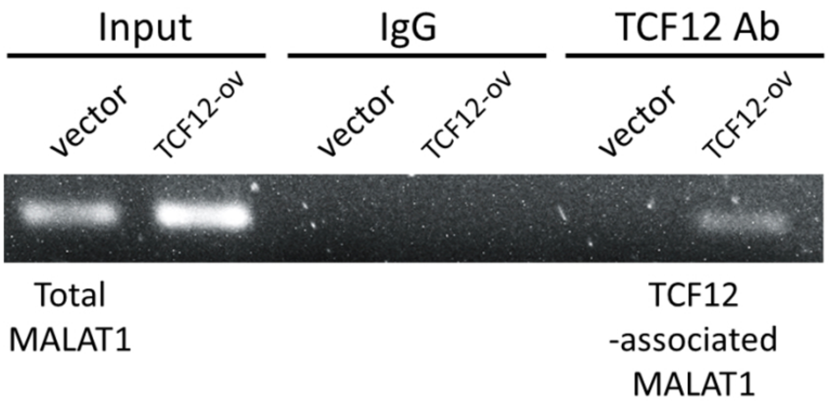

Supplementary Figure S1. Validation of MALAT1 as a TCF12-associated lncRNA. Anti-TCF12 immunoprecipitations were conducted from the lysates isolated from control (pcDNA3) and TCF12-overexpressing (TCF12-ov) SW480 cells. The immunoprecipitates were further subjected to RNA extraction and RT-PCR for detecting MALAT1 levels. Etopic TCF12 overexpression in SW480 cells also increased cellular MALAT1 level correspondingly.

A Univariate-Cox (RFS)

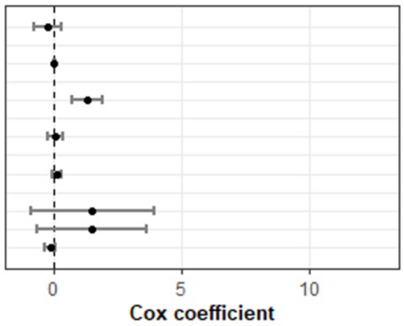

	Coef	lower	upper	p-value
genderMALE	-0.241	-0.800	0.319	0.400
age_at_initial_pathologic_diagnosis	0.027	0.000	0.054	0.051
Meta	1.305	0.706	1.904	0.000
TCF12	0.043	-0.265	0.351	0.784
MALAT1	0.101	-0.084	0.287	0.284
TCF12	1.508	-0.917	3.933	0.223
MALAT1	1.496	-0.630	3.622	0.168
TCF12•MALAT1	-0.135	-0.341	0.071	0.200

TCF12 × MALAT1

B Multivariate-Cox (RFS)

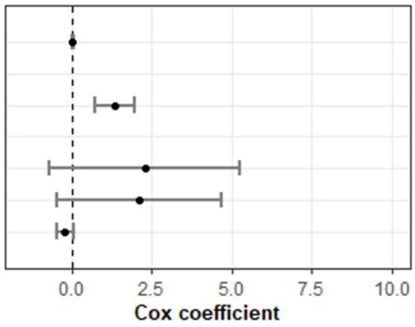

	Coef	lower	upper	p-value
Age	0.031	0.005	0.057	0.021
Meta	1.357	0.748	1.965	0.000
TCF12	2.278	-0.677	5.232	0.131
MALAT1	2.119	-0.449	4.688	0.106
TCF12•MALAT1	-0.200	-0.449	0.050	0.117

TCF12 × MALAT1

Supplementary Figure S2. Cox regression analyses of the association of TCF12–MALAT1 alliance with the relapse-free survival (RFS) of CRC patients. (A) Univariate Cox regression analyses of variates including gender, age, metastasis, TCF12 mRNA expression level, MALAT1 expression level, and the interaction term of TCF12 mRNA and MALAT1 expression levels (designated as "TCF12×MALAT1"). The TCF12×MALAT1 was represented as three covariates in a multivariate Cox regression analysis: TCF12 mRNA expression level (designated as "TCF12"), MALAT1 expression level (designated as "MALAT1"), and the multiplication of TCF12 mRNA and MALAT1 expression levels (designated as "TCF12•MALAT1". The data reveal that TCF12 mRNA expression and MALAT1 expression under an alliance rather than their alone had trend to associate with the poorer RFS of CRC patients. (B) A multivariate Cox regression analysis of covariates including age, metastasis, and TCF12×MALAT1.

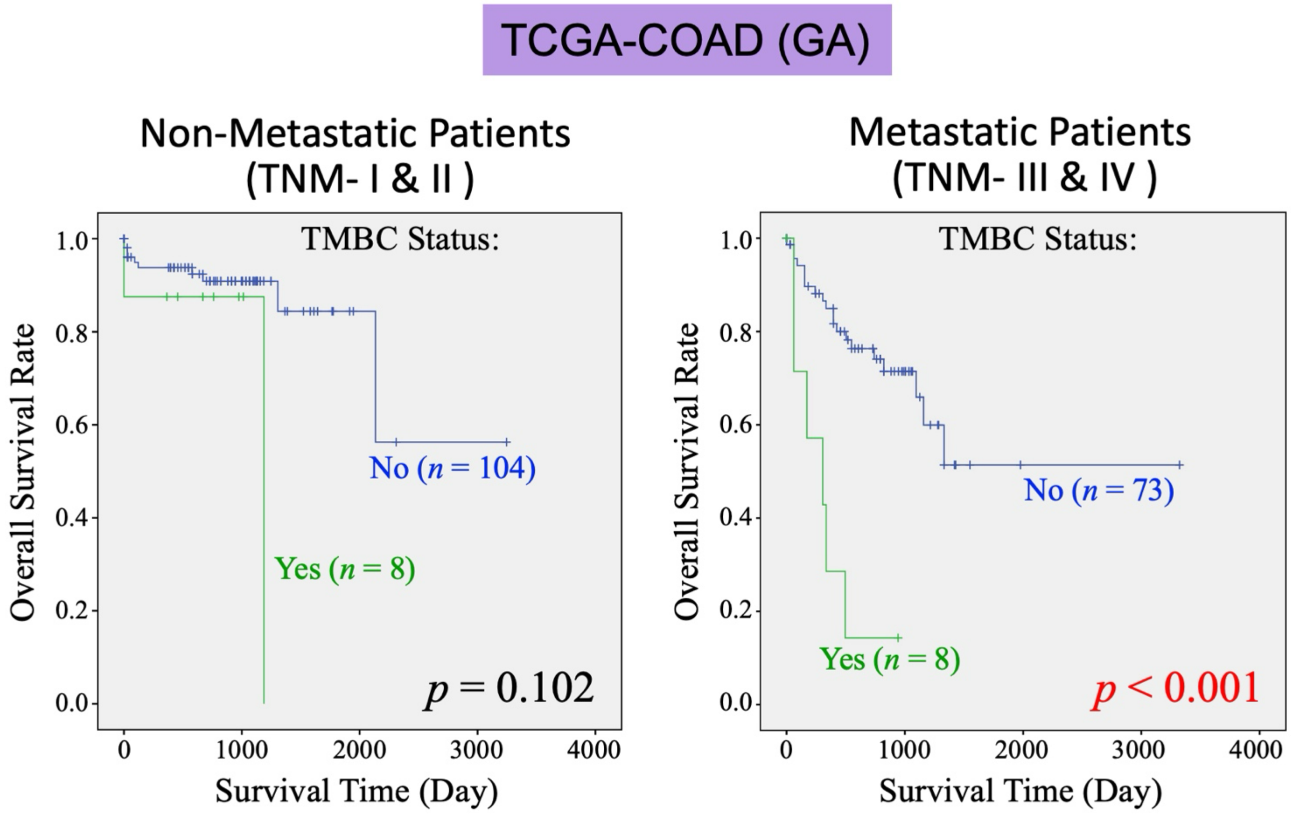

Supplementary Figure S3. Kaplan-Meier OS curves of non-metastatic (left panel) or metastatic (right panel) patients of the TCGA-COAD (GA) dataset, who were further divided into subgroups based on the patients with or without TMBC Pattern. The data reveal that the patients with TMBC pattern exhibited worse OS outcomes regardless of metastasis.
